# Clustering, Pathway Enrichment, and Protein-Protein Interaction Analysis of Gene Expression in Neurodevelopmental Disorders

**DOI:** 10.1155/2018/3632159

**Published:** 2018-11-27

**Authors:** Ruchi Yadav, Prachi Srivastava

**Affiliations:** Amity Institute of Biotechnology, Amity University Uttar Pradesh, Lucknow Campus, Lucknow 226028, UP, India

## Abstract

Neuronal developmental disorder is a class of diseases in which there is impairment of the central nervous system and brain function. The brain in its developmental phase undergoes tremendous changes depending upon the stage and environmental factors. Neurodevelopmental disorders include abnormalities associated with cognitive, speech, reading, writing, linguistic, communication, and growth disorders with lifetime effects. Computational methods provide great potential for betterment of research and insight into the molecular mechanism of diseases. In this study, we have used four samples of microarray neuronal developmental data: control, RV (resveratrol), NGF (nerve growth factor), and RV + NGF. By using computational methods, we have identified genes that are expressed in the early stage of neuronal development and also involved in neuronal diseases. We have used MeV application to cluster the raw data using distance metric Pearson correlation coefficient. Finally, 60 genes were selected on the basis of coexpression analysis. Further pathway analysis was done using the Metascape tool, and the biological process was studied using gene ontology database. A total of 13 genes AKT1, BAD, BAX, BCL2, BDNF, CASP3, CASP8, CASP9, MYC, PIK3CD, MAPK1, MAPK10, and CYCS were identified that are common in all clusters. These genes are involved in neuronal developmental disorders and cancers like colorectal cancer, apoptosis, tuberculosis, amyotrophic lateral sclerosis (ALS), neuron death, and prostate cancer pathway. A protein-protein interaction study was done to identify proteins that belong to the same pathway. These genes can be used to design potential inhibitors against neurological disorders at the early stage of neuronal development. The microarray samples discussed in this publication are part of the data deposited in NCBI's Gene Expression Omnibus (Yadav et al., 2018) and are accessible through GEO Series (accession number GSE121261).

## 1. Introduction

### 1.1. Neuronal Development Disorder

Neurogenesis is a process of generating new and functional neurons from neuronal precursors known as NSC (neuronal stem cells) [1, 2]. Functional neurons are generated at the embryonic stage at different stages of development throughout life [3, 4]. With rapid advancement in techniques and curiosity to understand neuronal diseases at the development stage, researchers have explored a wide area of neuronal development diseases and their causes [5–8]. Neuronal stem cells have two major features that are regeneration capacity, that is, ability of self-renewal by process of cell division, and differentiation capacity, that is, process of generating new and specialized cell types [9]. Developed neurons do not carry dendrites and axons, but they play an important role to receive and send signals to other neurons [10]. Significant development has been made to identify genes that are involved in neuronal diseases at the developmental stage [11]. It is important to study different stages of nervous system development and to identify abnormalities that can arise from improper development of brain at its early stage [12]. Significant contribution has been made by scientists to identify neuronal disorders that occur at the early stage of development [13]. Neuronal disorders include abnormalities associated with intellectual disability, attention deficit hyperactivity disorder (ADHD), and cognitive skills disorders, like dyslexia and dysgraphia, and language development disorders like expression disorder [14–18]. Scientific evidence shows that neurological disorders can be identified at the early stage by the first week or month of a lifecycle [19–21]. It is important to identify which genes are crucial and result in neurological disorders.

We have used high-throughput microarray experiment to identify genes that are involved in the early stage of neurodevelopment. Our aim was to identify genes that were expressed when stem cells were exposed to MCP (monocrotophos), a neurotoxicant, and to evaluate the effective role of resveratrol (RV) and nerve growth factor (NGF) as the neuroprotectant.

### 1.2. Resveratrol Clinical Perspectives

Resveratrol is a natural phenol and phytoalexin produced naturally by several plants in response to injury [22]. There is exponential evidence since 1939 in the literature that resveratrol is a promising natural compound for prevention and treatment of a wide range of human diseases [23]. Resveratrol is also reported to be effective against neuronal cell dysfunction and cell death, Huntington's disease and Alzheimer's disease [24–27]. Molecular studies show that resveratrol is associated with an induction of genes for oxidative phosphorylation and mitochondrial biogenesis [28]. Effect of resveratrol is known to extend lifespan, and it impacts mitochondrial function and metabolic homeostasis [29]. In the current work, we have mapped effectiveness of resveratrol against injured neurodevelopment samples. In this study, four samples were prepared (control, resveratrol, NGF, and RV + NGF). Datasets of prepared samples were taken to investigate the neuroprotective role of resveratrol against exposure of monocrotophos. In silico expression analysis of different datasets is done to identify genes that are coexpressed.

### 1.3. Microarray Data Analysis

Microarrays provide a rich source of data on the molecular mechanism of cell function. Each microarray reports expression of thousands of mRNAs [30]. Virtually, almost every human disease is being studied using microarrays experiment, with the aim of finding the novel genes involved in diseases and disease markers and to identify drug targets [31]. Bioinformatics analysis plays an important part of processing the information, embedded in large-scale expression profiling studies, and for laying the biological interpretation of high throughput microarray data [32]. A basic yet challenging task in the analysis of microarray gene expression data is the identification of changes in gene expression that are associated with particular biological conditions [33, 34]. Careful statistical design and analysis are essential to identify genes involved in each biological condition.

A standard workflow is required to utilize computational tools at various steps of microarray analysis. This paper also describes use of different bioinformatics tools for quality control, normalization, coexpression, annotation, and pathway and protein-protein interaction analysis.

### 1.4. Clustering and Coexpression Analysis

Clustering is a method to identify genes that are coexpressed in each biological condition [35]. Clustering methods uses a distance measure (e.g., Euclidean metric) to compare expression values of pairs of genes for each experiment [36]. When the distance between a pair of genes is small, then the two genes might be clustered. Clusters are analyzed to identify genes that are coexpressed and coregulated.

### 1.5. Biological Annotation and Interpretation

After extensive analysis of microarray data, one needs to annotate Affymetrix IDs for its significance. Annotation reveals the biological significance of genes like its molecular pathway, diseases involved, gene ontology, and so on [37]. Careful exploration is required to identify genes that are expressed in each condition of microarray experiment. Pathway and process enrichment is a crucial part of annotation, as it leads to the identification of set of genes that are involved in the same pathways [38]. Pathways analysis also highlights the set of proteins that interact with each other; this information is used to categorize protein interaction partners and to study protein-protein interaction network [39].

## 2. Materials and Methods

### 2.1. Microarray Data

The MSCs (mesenchymal stem cells) were used to study effect of monocrotophos (MCP) and repairing capability of resveratrol and nerve growth factor. MSCs were exposed to RV, NGF, and RV + NGF, respectively. In total, four samples were generated to identify genes that were coexpressed at the neuronal development stage. Affymetrix gene chip platform (Prime view.CDF) was used to identify gene expression using four samples as described in Table 1.

### 2.2. Microarray Data Analysis and Annotation

**Figure 1:**
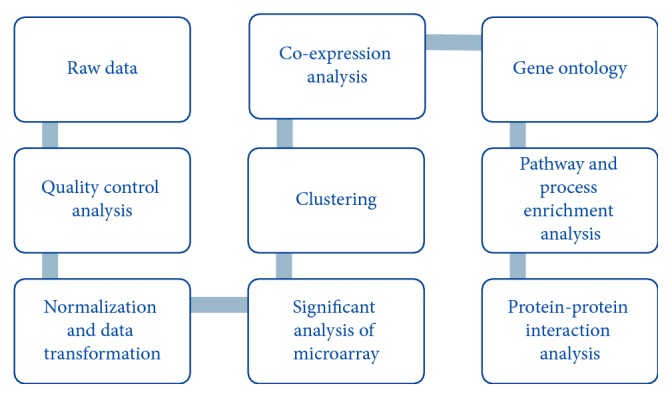
Workflow used for microarray data analysis and annotation.

Computational software and tools were used to identify genes that are coexpressed.  Figure 1 shows the workflow used for microarray data analysis and annotation. Raw files were used, i.e., chip electronic file (CEL) and chip description file (CDF) for quality control analysis. R and Bioconductor, Affy package, was used for data normalization and data transformation. Gene expression matrix was generated form Affy package, using RMA (robust multiarray average).

Significant analysis of microarray (SAM) [40] and clustering were done using MeV application [41]. The clustering method was used to cluster significant genes obtained from the SAM method. For clustering, the distance metric Pearson correlation coefficient was used, using parameter of k–means algorithm, number of cluster 10, and number of iteration 50. Coexpressed genes were identified by analyzing each ten clusters.

### 2.3. Pathway Enrichment and Protein-Protein Interaction Analysis

Coexpressed genes identified from clustering analysis were further annotated for biological intervention and pathway analysis. The list of coexpressed genes was searched against pathway and GO database using the Metascape tool (http://metascape.org) [42]. Each gene was studied for its pathway and process enrichment score for statistical significance of genes in each biological process. Genes were also clustered according to their pathways. Relationship between genes was also identified by using the network map generated from the Metascape tool and visualized in the Cytoscape tool [43].

Protein-protein interaction analysis is carried out by different protein interaction databases like BioGrid, InWeb_IM, and OmniPath by using the Metascape tool. Molecular complex detection (MCODE) algorithm was used by the Metascape tool to identify densely connected network of protein-protein interaction [44].

## 3. Results

### 3.1. Quality Control Analysis

**Figure 2:**
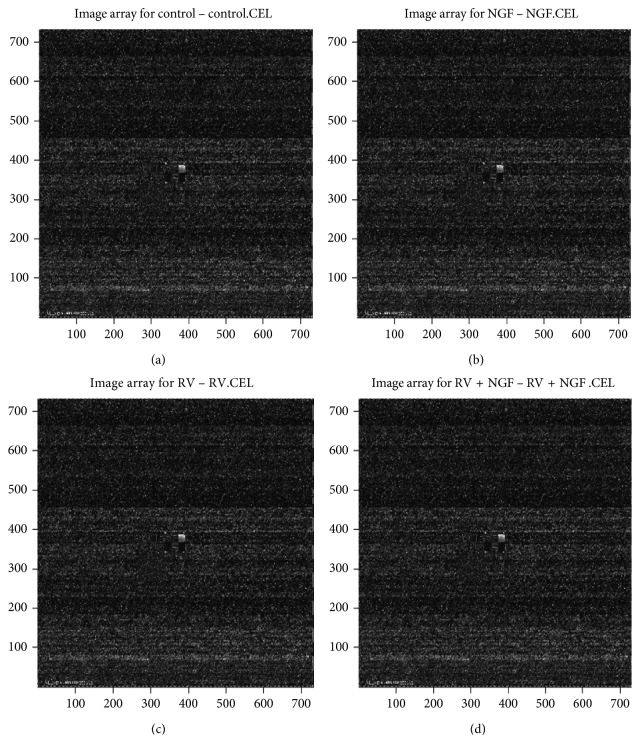
Affymetrix chip image: (a) control sample, (b) NGF sample, (c) RV sample, and (d) RV + NGF sample.

Quality control (QC) is an important aspect of examining microarray data, before any statistical analysis to be done. QC analysis was done using Affy package of R and Bioconductor [45].  Figure 2 shows the chip image of four samples: (a) control sample, (b) NGF sample, (c) RV sample, and (d) RV + NGF sample; it signifies there is no error in chips of all four samples, and they can be used for data exploration and analysis.

### 3.2. Data Normalization and Transformation

**Figure 3:**
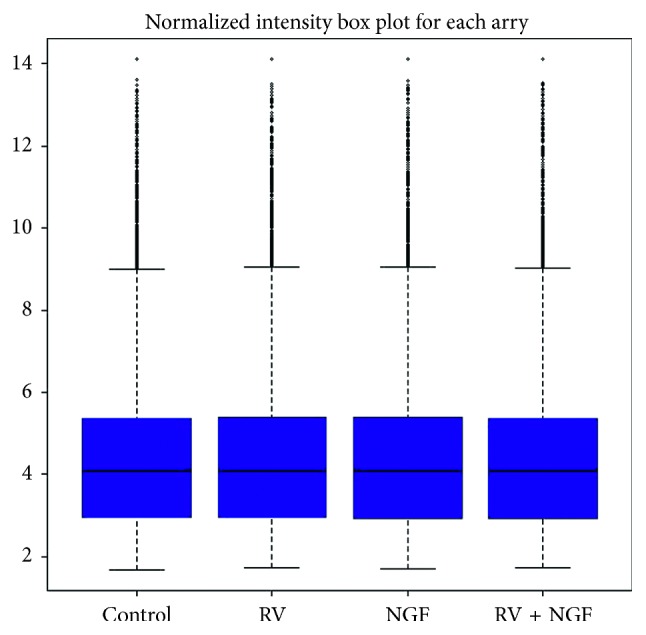
Box plot of microarray samples after normalization.

Finding biologically relevant answer from microarray experiment is a primary need of any microarray experiment. Variations in gene expression should be biologically not from any source of errors like biasness in dyes, lasers, samples, and chip spotting during microarray experiment [46]. To analyze microarray data, one needs to remove these biasness and errors in microarray experiment. Normalization is a method to remove these systematic errors that affect gene expression measures [47]. After QC analysis, normalization was done using Affy package of R and Bioconductor. We have used the RMA method of normalization.  Figure 3 shows the box plot of four samples after data normalization. Box plot shows statistical values like mean or median and variations between samples [48].  Figure 3 shows that means of all four samples are at position. Data were transformed to logarithm base 2 value of the expression ratio, and expression matrix was written, for further statistical analysis and comparisons.

### 3.3. Clustering and Coexpression Analysis

**Figure 4:**
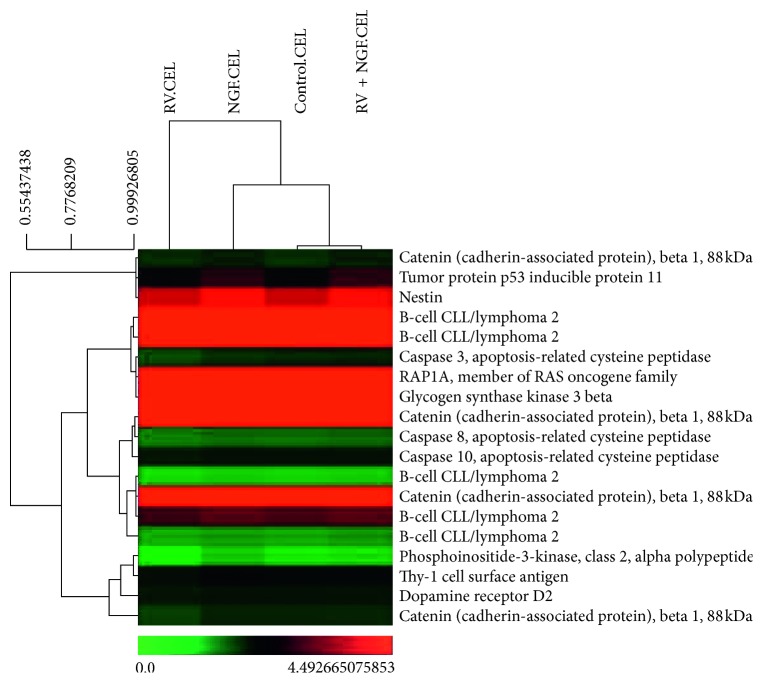
Clustering result of significant genes.

Significant analysis of microarray (SAM) was done to identify the number of genes that were statistically significant. Out of 49,495 genes, 49,022 genes were found to be insignificant and 473 genes were statistically significant. K-means clustering was done on significant genes with parameter of *k* = 10. Ten clusters were generated and studied for coexpression of genes.  Figure 4 shows cluster 1 (only one cluster is shown but all ten clusters were studied for coexpression analysis) which shows coexpression of Tp53 and B-cell cll/lymphoma2 gene. In addition, Caspase-8, Caspase-10, and dopamine receptor are also coregulated.

Analysis of all ten clusters results in identification of coexpressed genes. Rigorous analysis of clustering shows that 60 genes were coexpressed (AKT1, BAD, BAX, BCL2, BDNF, CASP3, CASP8, CASP9, MYC, PIK3CD, MAPK1, MAPK10, and CYCS). These genes were used for gene ontology, biological function, and pathway analysis. Descriptions including function of these 60 genes were shown in pathway and process enrichment analysis.

### 3.4. Pathway and Process Enrichment Analysis

Coexpressed genes that were clustered in the clustering step were used for biological annotation and interpretation. The Metascape tool was used to study pathway and process of these 60 genes. Protein-protein interaction network was constructed to identify more proteins that have similar function and belong to same pathway. 60 genes were further clustered into 20 groups on the basis of their enrichment score (enrichment score is the score between observed count and expected count by chance) [49].

**Figure 5:**
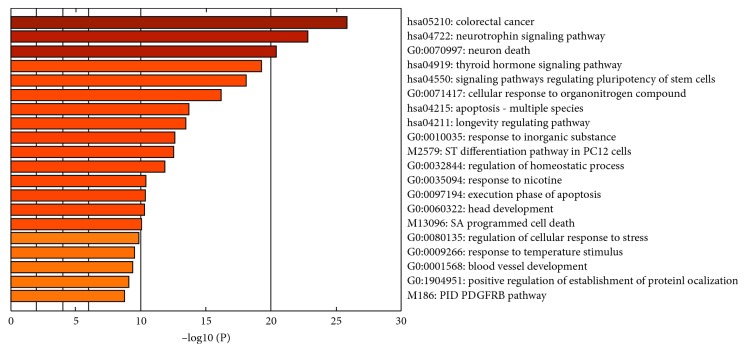
Heat map of enriched terms across input gene lists, colored by *p* values.

In each cluster, one term represents the cluster that is most statistically significant [42].  Figure 5 shows the heat map of enriched terms colored by the *p* value. Pathway enrichment analysis shows that most of the genes were involved in colorectal cancer, neurotrophin signaling pathway, neuron death, and thyroid hormone signaling pathway. Other clusters indicate genes that were involved in cellular response to organonitrogen compounds, response to nicotine, and head development. Genes that belong to these clusters were further studied in detail for function and pathway analysis.

Top 5 clusters are shown in Table 2; count is the number of genes in each cluster; percentage is the total gene ontology provided in list of genes; Log10(P) is the log base 10 value; and Log10(q) is the log base 10 adjusted *p* value [42].

Pathway enrichment shows that neuronal development genes are involved in colorectal cancer, neuron death, and other diseases like leukemia and sclerosis [50]. Genes AKT1, BAD, BAX, BCL2, CASP3, CASP8, CASP9, MYC, PIK3CD, MAPK1, MAPK10, and CYCS are commonly expressed in the cluster of colorectal cancer, neuronal signaling pathway, neuronal death, amytrophic lateral aclerosis, and tuberculosis [51]. Further proteins are identified that show interaction with these proteins on the basis of protein-protein interaction study.

### 3.5. Protein-Protein Interaction Enrichment Analysis

**Figure 6:**
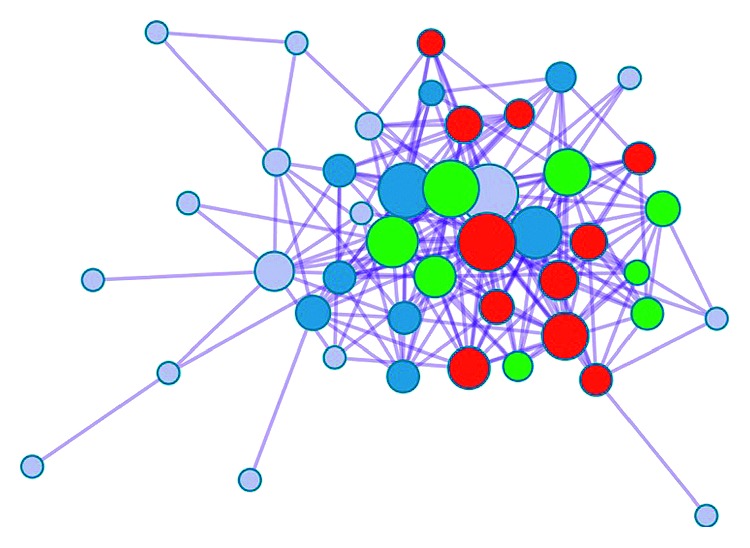
Protein-protein interaction network.

Protein-protein interaction (PPI) enrichment was done among the list of genes that were clustered in pathway and process enrichment analysis. The Metascape tool predicts PPI network by comparing it with protein interaction databases (BioGrid, InWeb_IM, and OmniPath) [42]. PPI is made between proteins having physical interactions, and PPI network is further subclustered on the basis of the *p* value score.  Figure 6 shows the PPI map between the set of input genes. Three best scoring genes by the *p* value are identified; these proteins define the functionality of PPI network. Best scoring genes belong to apoptosis (hsa04210) [52], colorectal cancer (hsa05210) [53], and hepatitis B (hsa05161) [54]. PPI network represents involvement of neuronal development genes in diseases like cancer.

**Figure 7:**
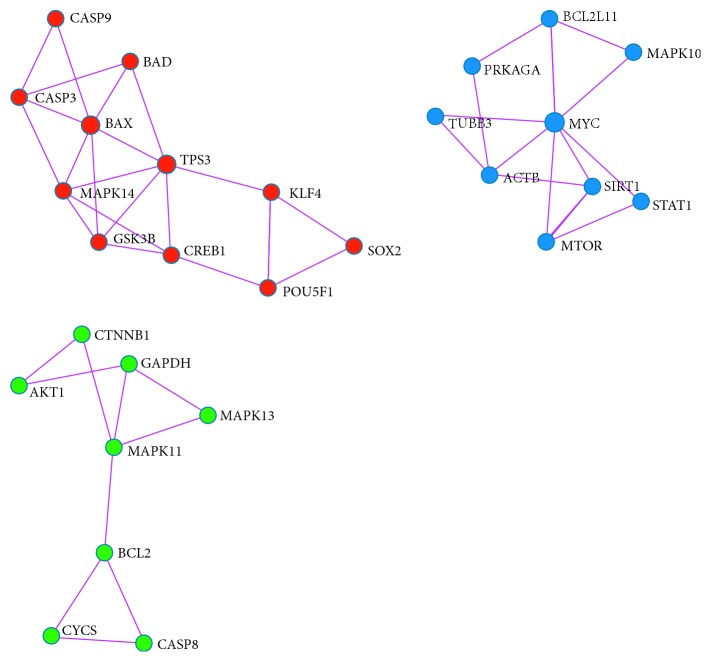
MCODE protein-protein interaction network. Colors show the different components of MCODE (red color: MCODE 1, blue color: MCODE2, and green color: MCODE 3).

The molecular complex detection (MCODE) method was applied to identify closely related protein from PPI network. The MCODE algorithm subclustered PPI network into 3 subclusters.  Figure 7 shows MCODE components (red, blue, and green as MCODE 1, 2, and 3). Three dense PPI were made and detail of each cluster is given in Table 3. MCODE prediction validates the results of clustering as previously shown in Figure 4. The same set of proteins was identified by MCODE algorithm as predicted by clustering using the MeV tool. These proteins have the same GO and pathway.

Cluster analysis of MCODE components is done, and details of proteins involved in each cluster and their corresponding pathways are shown in Table 3. Cluster 1 includes proteins CASP3, CASP9, BAX, TP53, BAD, GSK3B, POU5F1, MAPK14, CREB1, SOX2, and KLF4. Gene ontology data show that these proteins are associated with amyotrophic lateral sclerosis (hsa05014) [55], colorectal cancer (hsa05210) [56], and positive regulation of neuron death (GO: 1901216) [57].

Cluster 2 genes are mentioned in Table 3. GO analysis shows that these proteins belong to thyroid hormone signaling pathway (hsa04919) [55], diseases of signal transduction (R-HSA-5663202) [56], and pathways in cancer (hsa05200) [57]. Cluster 3 proteins are involved in pathways of toxoplasmosis (hsa05145) [55], tuberculosis (hsa05152) [56], and fluid shear stress and atherosclerosis (hsa05418) [55].

MCODE cluster and cluster of MeV software show that some genes were commonly expressed and were coregulated. AKT1, BAD, BAX, BCL2, BDNF, CASP3, CASP8, CASP9, MYC, PIK3CD, MAPK1, MAPK10, and CYCS genes are coregulated. PPI analysis identifies other proteins that have interaction with abovementioned proteins. These proteins are important in neuronal differentiation, and regeneration proteins like ACTB, GSK3B, CREB1, and CTNNB1 have physical interaction with coexpressed proteins [58]. Table 3 also gives the information about proteins and association with diseases. Analysis of disease associated with proteins highlights that some proteins belong to different classes of cancers. 12 proteins (CASP3, CASP9, BAX, TP53, BAD,GSK3B, MTOR, BCL2L11, SIRT1, CASP8, AKT1, and C TNNB1 proteins) are involved in diverse types of cancers like lung cancer, breast cancer, ovarian cancer, colorectal cancer, and leukemia [59].

While other proteins (GSK3B, POU5F1, MAPK14, CREB1, SOX2, KLF4, PRKACA, MAPK10, STAT1, ACTB, TUBB3, MYC, GAPDH, AKT1, and CTNNB1) are related with process of aging, neuronal diseases, cardiovascular diseases, abnormal brain development, mental retardation, schizophrenia, and mycobacterial and viral infections [60–62].

Key findings of the pathway and disease association study are identification of proteins involved in neurological diseases and also expressed at the early stage of neuronal development. SOX2 protein was expressed in optic nerve hypoplasia and abnormalities of the central nervous system [63], STAT1 was expressed during mycobacterial and viral infections [64], TUBB3 was related with fibrosis and cortical dysplasia and brain deformities, AKT1 was expressed in breast cancer, colorectal cancer, ovarian cancer, and schizophrenia [65] and CTNNB1 was expressed in colorectal cancer, hepatocellular carcinoma, ovarian cancer, and mental retardation [66]. The study shows that proteins (SOX2, STAT1, AKT1, and CTNNB1) can be used as markers for neurological disease at the early stage of neuronal development, and they can be potential drug targets for therapeutic development.

## 4. Conclusion and Discussion

Microarray experiment is designed to investigate the genes that are expressed at the early stage of neuronal development. Neurodevelopmental microarray gene expression data are used to identify genes that are expressed in neuronal disorders, at its initial stage of progress [67]. Four samples were prepared, viz, control, resveratrol, nerve growth factor, and RV + NGF and hybridized to Affymetrix chip (Prime view). Gene expression matrix was constructed, and computational analysis was done. The protocol is designed to study biologically significant genes. Microarray data analysis workflow includes quality control, data normalization, clustering, pathways enrichment, and PPI study. Clustering analysis identifies genes that are coexpressed. These sets of coexpressed genes are used for pathway and process enrichment analysis. Gene ontology and pathway study reveal proteins that share common pathways and function. Further protein-protein interaction network is constructed to identify more number of proteins, which have physical interaction with coexpressed proteins. PPI network is subclustered to predict closely related proteins. Gene ontology information of these proteins is used to identify function and disease associated with proteins. 12 proteins CASP3, CASP9, BAX, TP53, BAD, GSK3B, MTOR, BCL2L11, SIRT1, CASP8, AKT1, and CTNNB1 proteins are predicted that are involved in various types of cancers like lung cancer, breast cancer, ovarian cancer, colorectal cancer, and leukemia [60, 61, 62]. Some proteins like SOX2, STAT1, AKT1, and CTNNB1 proteins are associated with neurological disease like abnormal brain development, mental retardation, schizophrenia, and mycobacterial and viral infections [63–66]. These genes can be used as markers for neurological disease, for detection of abnormalities at the early stage of neuronal development [67]. Predicted proteins can also act as potential drug targets for the drug development process. Further work is required for wet lab verification of predicted genes that are expressed in neurological disorders and express at the developmental stage. More research is required in the field of neurodevelopmental biology to identify neurological abnormalities at its budding stage. This paper also highlights the importance of microarray experiment in understanding the neurological diseases and methodology to study various outcomes of gene expression data, like coexpression analysis, pathway and process identification, and protein-protein interaction network study.

## Figures and Tables

**Table 1:** Four samples that were used for microarray gene expression analysis.

S.no	Samples	Description	Raw files
1	Control	Control sample	Control.CEL
2	RV	MSCs exposed to RV	RV.CEL
3	NGF	MSCs exposed to NGF	NGF.CEL
4	RV + NGF	MSCs exposed to combined RV + NGF	RV + NGF.CEL

**Table 2:** Pathway and process enrichment analysis.

S.no	GO	Category	Description	Count	%	Log10 (P)	Log10 (q)
1	Hsa05210	KEGG pathway	Colorectal cancer	15	25	−25.7	−21.4
2	Hsa04722	KEGG pathway	Neurotrophin signaling pathway	16	26.6	−22.8	−19.1
3	Go: 0070997	Go biological processes	Neuron death	19	31.6	−20.3	−16.9
4	Hsa04919	KEGG pathway	Thyroid hormone signaling pathway	14	23.3	−19.2	−15.9
5	Hsa04550	KEGG pathway	Signaling pathways regulating pluripotency of stem cells	14	23.3	−18.0	−14.9

**Table 3:** Cluster details of MCODE protein-protein interaction and pathways.

Cluster	Symbol	Pathways	Diseases
1	CASP3	Caspase-3	Cancer
	CASP9	Caspase-9	Aging, cancer, chemdependency
	BAX	BCL2-associated X and apoptosis regulator	Colorectal cancer, somatic, T-cell acute lymphoblastic leukemia
	TP53	Tumor protein p53	Breast cancer, colorectal cancer, hepatocellular carcinoma, etc.
	BAD	BCL2-associated agonist of cell death	Cancer, hematological, infection
	GSK3B	Glycogen synthase kinase 3 beta	Cancer
	POU5F1	POU class 5 homeobox 1	Chemdependency
	MAPK14	Mitogen-activated protein kinase 14	Chemdependency
	CREB1	cAMP responsive element-binding protein 1	Histiocytoma
	SOX2	SRY-box 2	Optic nerve hypoplasia and abnormalities of the central nervous system
	KLF4	Kruppel like factor 4	Cardiovascular and metabolic diseases

2	MTOR	Mechanistic target of rapamycin	Aging, cancer, chemdependency
	PRKACA	Protein kinase cAMP-activated catalytic subunit alpha	Cushing syndrome
	MAPK10	Mitogen-activated protein kinase 10	Metabolic diseases
	STAT1	Signal transducer and activator of transcription 1	Mycobacterial and viral infections
	ACTB	Actin beta	Baraitser-winter syndrome 1 and dystonia
	BCL2L11	BCL2 like 11	Cancer, cardiovascular diseases
	SIRT1	Sirtuin 1	Aging, cancer, cardiovascular diseases
	TUBB3	Tubulin beta 3 class III	Fibrosis, cortical dysplasia, brain malformations
	MYC	v-myc avian myelocytomatosis viral oncogene homolog	Burkitt lymphoma

3	GAPDH	Glyceraldehyde-3-phosphate dehydrogenase	Aging
	CASP8	Caspase-8	Breast cancer, lung cancer
	AKT1	AKT serine/threonine kinase 1	Breast cancer, somatic, colorectal cancer, ovarian cancer, schizophrenia
	BCL2	BCL2, apoptosis regulator	Leukemia/lymphoma
	CYCS	Cytochrome c, somatic	Thrombocytopenia
	MAPK11	Mitogen-activated protein kinase 11	Unknown
	MAPK13	Mitogen-activated protein kinase 13	Unknown
	CTNNB1	Catenin beta 1	Colorectal cancer, hepatocellular carcinoma, ovarian cancer and mental retardation

## Data Availability

The microarray data used to support the findings of this study are included within the supplementary information file.

## Supplementary Materials

Supplementary Materials Gene expression matrix microarray raw files (CEL and CDF files) were used to make gene expression matrix using Affy package and RMA (robust multiarray average) method. This matrix file was used for further microarray analysis like clustering, pathway, and protein-protein interaction analysis. Pathway and protein-protein interaction result as generated from the Metascape tool is given. This file includes information about clustered formed within genes and network details with scores. Annotation and enrichment annotation file and pathway enrichment result as generated from the Metascape tool is given. Annotation file includes information about genes like gene symbol, gene description, GO biological process, protein function, and so on.
