# Supplementary material for: Clustering, Pathway Enrichment, and Protein-Protein Interaction Analysis of Gene Expression in Neurodevelopmental Disorders
Source: Adv Pharmacol Sci. 2018 Nov 27;2018:3632159. doi: 10.1155/2018/3632159 (PMC6288580; doi:10.1155/2018/3632159)
Supplement: Supplementary Materials — Gene expression matrix microarray raw files (CEL and CDF files) were used to make gene expression matrix using Affy package and RMA (robust multiarray average) method. This matrix file was used for further microarray analysis like clustering, pathway, and protein-protein interaction analysis. Pathway and protein-protein interaction result as generated from the Metascape tool is given. This file includes information about clustered formed within genes and network details with scores. Annotation and enrichment annotation file and pathway enrichment result as generated from the Metascape tool is given. Annotation file includes information about genes like gene symbol, gene description, GO biological process, protein function, and so on. [file 3632159.f1.zip › SUPPLEMETARY DATA.docx]

**SUPPLEMENTARY DATA**

1. Microarray gene expression file
2. MCODE result file
3. MCODE Pathway and protein-protein interaction network
4. Metascape annotation results
